# Designs of two randomized, community-based trials to assess the impact of influenza immunization during pregnancy on respiratory illness among pregnant women and their infants and reproductive outcomes in rural Nepal

**DOI:** 10.1186/s12884-015-0470-y

**Published:** 2015-02-20

**Authors:** James M Tielsch, Mark Steinhoff, Joanne Katz, Janet A Englund, Jane Kuypers, Subarna K Khatry, Laxman Shrestha, Steven C LeClerq

**Affiliations:** Department of Global Health, Milken Institute School of Public Health, George Washington University, Washington, DC USA; Global Health Center, Cincinnati Children’s Medical Center, Cincinnati, OH USA; Department of International Health, Johns Hopkins Bloomberg School of Public Health, Baltimore, MD USA; Seattle Children’s Hospital and Research Foundation, University of Washington, Seattle, WA USA; School of Medicine, University of Washington, Seattle, WA USA; Nepal Nutrition Intervention Project – Sarlahi, Kathmandu, Nepal; Department of Pediatrics and Child Health, Institute of Medicine, Tribhuvan University, Kathmandu, Nepal

**Keywords:** Influenza vaccination, Pregnancy, Early infancy, Influenza like illness, Influenza illness, Birthweight

## Abstract

**Background:**

Among the most important causes of illness and death in both pregnant women and their newborn infants are respiratory infections including influenza. Pregnant women in North America have a 4 to 5 fold excess rate of hospitalization compared to non-pregnant women. Rates of infant hospitalization associated with influenza are much higher than in their mothers. Fully half of children hospitalized for influenza in the US are in the age group 0–5 months, a group where no vaccine is licensed. Data on influenza are much fewer in low income countries where the risks of serious morbidity and mortality are much higher. A recent trial in Bangladesh suggested that influenza immunization in pregnant women could have important protective effects against influenza in both mothers and their infants. These trials were designed to provide additional evidence about the effect of influenza vaccination in pregnancy in settings where influenza may circulate for up to ten months/year.

**Methods/Design:**

We conducted a consecutive pair of community-based, placebo-controlled, randomized trials of influenza vaccination of pregnant women in a rural district in southern Nepal. Two trials were conducted to insure, as much as possible, the match of circulating strains with those included in the vaccine. Eligible women included all who were or became pregnant over a one year period. Each trial included a one year cohort of pregnant women who were individually randomized to the influenza vaccine available at the time of their enrollment or placebo. Exclusions included a history of allergy to vaccine components, prior influenza vaccine receipt, and for the second trial, participation in the first trial. Morbidity was assessed on a weekly basis for women throughout pregnancy and through 180 days post-partum. Infants were followed weekly through 180 days. Primary outcomes included: 1) incidence of influenza like illness in women, 2) incidence of laboratory confirmed influenza illness in infants, and 3) birthweight among newborn infants.

**Discussion:**

We have presented the design and methods of two randomized trials of influenza immunization of pregnant women.

**Trial registration:**

Clinicaltrials.gov: (NCT01034254).

## Background

Pregnancy and delivery are hazardous for mothers and infants in low resource regions, with rates of illness and death many times higher than in wealthier regions. Among the most important causes of these illnesses and deaths in both mothers and infants are respiratory infections including influenza [1]. The best estimates of the clinical significance of complications of respiratory disease during pregnancy are based on indirect data from developed countries [2]. Remarkably few data exist on the prevalence or significance of laboratory-proven influenza infections in pregnant women and the outcomes of infection with influenza in the woman and her fetus, particularly in developing countries [3]. The increased risk of influenza in pregnant women is related to physiologic changes including increases in blood volume, decreases in lung capacity, and attenuation of cell-mediated immune responses [4]*.* Excess mortality with rates approaching 50% were reported in pregnant women during the 1918–19 influenza pandemic, some of the highest rates of any patient group. Influenza was also a leading cause of maternal mortality during the 1957 pandemic [5] and an important cause of maternal morbidity and mortality during the 2009–10 influenza A/H1N1 pandemic [6]. The impact of influenza on the fetus when influenza is contracted during pregnancy is less clear, although cases of stillbirths and fetal deaths have been reported [7] and increased rates of prematurity were documented in the most recent pandemic [8].

A case–control study of US women showed that healthy pregnant women have a four-fold increased rate of hospitalization during influenza season compared to post-partum controls [9]. This indirect evidence has been validated by several studies in the United States and, most recently, by studies in Canada that show a five-fold seasonal increase in the risk of hospitalization among pregnant women [10-12]. Limited data exist documenting influenza infection in pregnant women using laboratory methods, although a study from Britain showed serologic evidence of influenza infection during pregnancy in 11% of 1,659 pregnant women, associated with an overall 43% increased rate of complications in the delivery, postpartum, or newborn infant periods, when compared to uninfected controls [3]. A study of over 6 million hospital admissions of pregnant US women showed that hospitalization with a respiratory illness during pregnancy in influenza season was associated with 4-fold increased risk of preterm delivery and cesarean section, and a 2.5-fold increase in reports of fetal distress, compared to hospitalizations without a respiratory illness [13]. Because of the increased risk of hospitalization, and proven safety of influenza vaccine in pregnant women, routine immunization of all pregnant women during influenza season has been recommended in the US since 1997, and by WHO since 2005.

US infants and children have relatively high rates of illness and hospitalization for influenza infection, despite increasing availability of laboratory diagnostics and the availability of licensed influenza vaccine for this age group [14]. The high rates of illness and hospitalization rates of ~1/1000 prompted the US recommendations in 2004 for universal immunization of infants from 6–59 months. More recently, it has become clear that US infants from 0–5 months of age have a substantial burden of influenza, with hospitalization rates of 0.8 to 1.2% [7,14]. These rates of young infant hospitalization are higher than in other recognized high-risk groups for influenza, such as adults over 60, and much higher than in their mothers. Of all US children under five years of age hospitalized for proven influenza, approximately 50% are in the age group 0–5 months [15], an age group where licensed influenza vaccine is not available. In addition, medically attended illness rates attributable to influenza are 50–100 fold higher than the influenza hospitalization rates in this young infant group [9,14].

Maternal immunization to protect both mothers and their young infants is currently recommended to prevent neonatal tetanus (in limited resource regions) and influenza (in the US, UK, and Canada) [15]. While maternal immunization is a safe and effective strategy with potential to advance both Millennium Development Goals 4 and 5 (child and maternal mortality, respectively), this approach remains underutilized in limited resource regions to prevent illness in mothers and infants [16]. Because the delivery system of antenatal immunization is in place for tetanus toxoid in many low-resource regions, the maternal immunization strategy is programmatically feasible and sustainable for other vaccines [17].

Vaccines with potential to reduce illness of both mothers and infants include influenza, pertussis, and pneumococcal vaccines. These vaccines are known a) to be safe in pregnant women, b) to elicit functional antibody during the period of high risk in mothers, which can then be transferred to the fetus c) deliverable in existing antenatal tetanus toxoid programs, and d) relatively low cost [12]. A recent comparative trial of trivalent inactivated influenza vaccine (TIV) and pneumococcal polysaccharide vaccines in pregnant women in Bangladesh showed substantial protection of both mothers and their infants by maternal immunization with TIV. Maternal influenza immunization reduced laboratory-proven influenza in their infants by 68% from birth to six months of age, and also reduced episodes of maternal influenza-like illnesses (ILI) by 35% [18]. In addition, there were significant improvements in birthweight associated with receipt of influenza vaccine in pregnancy [19]. However, the Bangladesh trial was relatively small and not designed to specifically address the impact of maternal influenza vaccine. Therefore, additional studies to answer this question in a variety of contexts are necessary to inform policy making for immunization programs.

## Methods

### Design

Two, consecutive, individually randomized, placebo controlled trials of influenza immunization during pregnancy were conducted in southern Nepal. Each trial recruited a population-based cohort of pregnant women who were between 17 and 34 weeks gestation over a one year period. The choice of two independent cohorts was used to insure, as much as possible, there would be a match between circulating strains of influenza and that year’s vaccine strains. Two additional substudies were also conducted during these randomized, controlled trials. The first was conducted in a subsample of mother/infant pairs in both trials and assessed the degree to which influenza vaccine produced vaccine-specific antibody in the mothers and the level of antibody transferred to her infant as measured in umbilical cord blood and breast milk. The second was done in a subsample during the first trial and determined the impact of maternal influenza immunization on indirect protection from ILI and laboratory confirmed influenza disease among other household members.

### Study population

The study population for these trials included all married women between 15 and 40 years of age who were identified as pregnant with gestational age between 17 and 34 weeks during a 12-month period in 9 Village Development Committees (VDCs) of Sarlahi District in the terai region of southern Nepal. The terai is the southernmost region of Nepal, lies along the border with India, and is part of the flood plain of the Ganges River and its tributaries that drain from the Himalayas to the Bay of Bengal. Sarlahi District is low and flat (approximately 131 m elevation) with high population density (635/km^2^) [20]. It is typical of the entire Indo-Gangetic plain with a population of over one billion. It is predominately an area of traditional, rural, Hindu culture. The population is mostly peasant farmers (58%) or laborers (26%) and their families and is considered a poor area in Nepal with an estimated poverty gap of 0.47 in 2010 [20].

Pregnant women were identified through a baseline survey of all households at the start of the first trial and subsequently through 5-weekly follow-up of households where women of reproductive age resided. Pregnancy was documented using commercially-available urine pregnancy tests. Only one pregnancy per woman was enrolled. The following pregnant women were excluded in both trials:

- Women who did not intend to deliver their child within the 9 VDCs in the study area.
- Women who had already received the current influenza vaccine.
- Women who were allergic to any component of the vaccine. This exclusion was mostly related to allergies to eggs.
- Women who refused to provide consent.
- Women who were later than 34 weeks gestation at the time their pregnancy was identified by the study.
- Women will be excluded from the analysis of primary outcomes if they deliver their child less than 2 weeks following receipt of the vaccine or placebo.

Two sequential annual cohorts of pregnant women were enrolled into independent trials.

In addition to the exclusions listed above, women who participated in the first trial were not eligible for the second trial.

- **Eligibility Phase 1:** Women who were pregnant and between 17 and 34 weeks gestation at the time of trial enrollment for the period April 25, 2011 through April 24, 2012 were eligible for participation in the first trial if they were not excluded based on the above mentioned exclusion criteria.
- **Eligibility Phase 2:** Women who were pregnant and between 17 and 34 weeks gestation at the time of trial enrollment for the period April 25, 2012 through April 24, 2013 were eligible for participation in the second trial if they did not meet exclusion criteria and if they had not participated in the first trial.

The date of trial enrollment for both phases was defined as the day they presented to the vaccination clinic and were randomized.

### Intervention

The intervention tested in these trials was year-round immunization of pregnant women between 17 and 34 weeks gestation with a commercially available, trivalent inactivated influenza vaccine licensed in Nepal. Vaxigrip vaccine (Sanofi Pasteur Ltd.) was used and purchased by the project from the Yetichem Group in Kathmandu, Nepal, which sourced the vaccine from Sanofi India, Mumbai. The vaccine was the current vaccine at the time of enrollment for an individual woman. That is, women were scheduled to receive the most up-to-date influenza vaccine at the time they were enrolled. After the regular change in vaccine, the vaccine was switched to the newly available vaccine. Table 1 shows the dates and formulations of the trivalent vaccines used throughout both study periods. The control group received placebo (saline injection).

**Table 1:** **Vaccines used by date**

**Vaccine formulation**	**Vaccine strains**			**Sanofi Pasteur Vaxigrip® lot no.**	**Dates of use**
	**A/H3N2**	**A/H1N1**	**B**		
**Northern 2010-2011**	Perth	California	Brisbane(V)	G7142	24 Apr 11 to 29 Jul 11
**Southern 2011**	Perth	California	Brisbane(V)	H9100	7 Aug 11 to 30 Dec 11
**Northern 2011-2012**	Perth	California	Brisbane(V)	H7099-1	1 Jan 12 to 31 May 12
				H7116-2	1 Jun 12 to 29 Jun 12
**Southern 2012**	Perth	California	Brisbane(V)	J7025-1	1 Jul 12 to 15 Oct 12
**Northern 2012-2013**	Victoria	California	Wisconsin(Y)	J7186-1	15 Oct 12 to 30 Dec 12
**Southern 2013**	Victoria	California	Wisconsin(Y)	J7154-4	30 Dec 12 to 27 Jan 13
				J7217-1	27 Jan 13 to 21 May 13
				K7021-3	21 May 13 to 3 Sept 13
				K7209-3	3 Sept 13 to 9 Sept 13

Women who were in their first trimester of pregnancy (<17 weeks) at the time of pregnancy identification had their enrollment delayed until after 17 weeks. In both groups, tetanus toxoid (TT) immunization status was assessed and if a woman failed to report a history of adequate immunization (i.e. 2 doses during the current pregnancy or 5 total prior doses during her reproductive years), TT was provided in the opposite arm at the same time as the study vaccine (influenza/placebo).

The timing of vaccine delivery differed in the two trials. In the first trial, women were enrolled and vaccinated as soon as they were identified as pregnant and were between 17 and 34 weeks gestation. In the second trial, after pregnancy identification, women were randomly assigned to a week of gestation for enrollment and vaccination that ranged from 17 weeks (or their gestational age at the time of pregnancy identification, which ever was later) to 34 weeks. Vaccine was administered by unmasked study nurses in clinics and homes; these nurses did not participate in the weekly illness assessment of the mother or infant. Mothers and other staff/investigators were masked. At the conclusion of the study, all women randomized to placebo were offered influenza vaccine.

Vaccine and placebo were transported from the supplier in Kathmandu to the field, and from the field study office to vaccination clinic locations in monitored coolers with continuous temperature tracking. Any vaccine not kept within the manufacturer’s recommended temperature range prior to use was discarded.

### Outcomes

There were 3 primary and several secondary outcomes for these trials.

### Primary outcomes

1. The incidence of laboratory confirmed influenza illness episodes in the infant through 6 months of age.
2. The incidence of low birthweight among newborn infants.
3. Incidence of influenza-like febrile illness (ILI) episodes of the mother during pregnancy and through 6 months postpartum.

Signs and symptoms were ascertained during weekly home visits from the time of maternal enrollment through 180 days following delivery. Every week, field staff visited the home and asked about a series of signs and symptoms for each day in the prior week. For both pregnant and post-partum women, these included fever, persistent cough, sore throat, nasal congestion, myalgia and diarrhea. For infants, these included high fever, cough, difficult or rapid breathing, rhinorrhea, wheeze, dripping ear, diarrhea, difficulty feeding, jaundice and receipt of immunizations. We also ascertained if care was sought for any illness and the type of provider consulted. If a woman or infant had signs/symptoms included in our definitions, a single sterile nasopharyngeal swab with a nylon flocked tip (Copan Diagnostics, Murrieta, CA) was inserted into a nare approximately one-half the distance between the external nostril and the nasal bridge and twisted 360° to obtain a mid-nasal respiratory specimen. It was then placed in Primestore molecular transport medium (Longhorn Vaccines and Diagnostics LLC, Bethesda, MD) and the shaft broken off prior to closing the tube. The test tube was stored at room temperature for up to one week before being transported to the local laboratory. In a subsample of participants, an additional nasal swab was collected from the other nare and placed in viral culture medium.

#### Definitions

- **Laboratory Confirmed Influenza in Infants:** An episode of respiratory illness (with one of more of the following: reported or measured fever (axillary temperature >38°C), cough, wheeze, difficult or rapid breathing, or draining ear plus a positive laboratory test for influenza from nasal swab(s).
- **Low birthweight (LBW) in newborn infants:** The internationally accepted definition <2500 g was considered to be LBW. Eligible weights were those measured within 72 h of delivery. A secondary analysis included a back calculation to the weight at the time of delivery for all infants based on a statistical model developed from longitudinal weight measures from newborns included in a previous study in this population.
- **Influenza-Like Illness in Mothers (ILI):** The CDC definition of ILI was used that requires reported or measured fever (axillary temperature >38°C) plus either cough or sore throat on one or more days.

Episodes of influenza-like illness were required to be separated by 7 or more symptom-free days. Laboratory-confirmed influenza for both mothers and infants is not simply a subset of ILI, as ILI required fever as part of the definition and respiratory samples were obtained using a broader definition. This broader clinical definition of signs and symptoms included in the definition for laboratory confirmed influenza reflects the need to identify cases of influenza with a lower severity of clinical signs and symptoms than that included with ILI.

### Secondary outcomes

1. The incidence of clinic visits and hospitalizations for mothers and infants. These were recorded on the weekly morbidity forms and linked to episodes of ILI or respiratory illness.
2. The incidence of ILI and influenza illness in family members. Definitions for ILI and laboratory confirmed influenza will be the same as for mothers and children. Definitions for adults and children 5 years or older will use the maternal definitions. Definitions for children less than 5 years will use the infant definitions.
3. The incidence of ILI in Infants.: We will use a modified CDC definition of influenza-like illness for the infants, including reported or measured fever (axillary temperature >38°C) plus cough, or draining ear, or difficulty breathing occurring on one or more days [2].
4. The incidence of laboratory-confirmed influenza in mothers. This required an episode of respiratory illness (reported or measured fever (axillary temperature >38°C) plus one or more of the following: cough, sore throat, runny nose, nasal congestion, or myalgia), in addition to a positive laboratory test for influenza from a nasal swab.
5. Gestational age. Defined as the difference between the date of birth and the date of the start of the woman's last menstrual period (LMP). LMP was assessed at the time pregnant women were identified. Preterm birth was defined as gestational age <37 completed weeks at the time of delivery.
6. Small-for-Gestational-Age. Infants born small-for-gestational-age were those infants born below the 10^th^ percentile of weight for gestational age [21].
7. Growth. Attained growth was measured at 6 months of age in all enrolled infants. Weight, length, and head circumference were measured and weight-for-age, length-for-age, and weight-for-length Z scores were calculated based on the WHO growth standards [22].
8. Causes of Febrile Illness: Causes of ILI and respiratory infections were defined based on virologic and pertussis assays taken from nasal swabs.

### Randomization

Pregnant women were individually randomized to either intervention or control at the time of enrollment. Enrollment was defined as the date on which the women was randomized and received her assigned “treatment”. In phase 1 (April 25, 2011 – April 24, 2012) vaccination was scheduled as soon as possible after 17 weeks gestation or at 17 weeks if the pregnancy was identified prior to 17 weeks. Randomization was stratified by VDC and gestational age (GA) (two strata 17–25 weeks, 26–34 weeks) in blocks of size 8. In phase 2 (April 25, 2012 - April 24, 2013), randomization was stratified by VDC and gestational age (GA) (two strata 17–25 weeks, 26–34 weeks) and in blocks of size 8. For both phases, individual envelopes were prepared with a sequential serial number on the outside of a sealed envelope (separate set of ordered envelopes for each VDC-Gestational Age stratum in both phases and the treatment assignment for that participant was printed on a piece of paper inside the envelope). When a woman of child-bearing age was identified as pregnant, she was given an appointment date to be formally enrolled and receive her assigned treatment. Upon arrival at the clinic, she had her eligibility reconfirmed and then was listed on a sequential vaccination receipt log specific to her VDC-Gestational Age stratum. Once in the vaccination room, the assigned sealed envelope was opened and she received the treatment (code A, B C, or D) found inside. At the clinic, vaccine and placebo were pre-coded and the appropriate vaccine was provided to the participant based on the code contained in her pre-numbered envelope.

### Data collection

The timing of data collection activities is outlined in Table 2 and described in more detail below.

**Table 2:** **Data collection schedule**

**Data items**	**Pregnancy identification**	**Vaccine delivery**	**Weekly during pregnancy**	**Monthly during pregnancy**	**Delivery**	**1 month**	**3 month**	**6 month**	**Weekly through 6 months post-partum**
Mother’s Demographic and SES Characteristics	X								
Mother’s Reproductive History	X								
Mother’s Anthropometry	X								
Maternal Vaccination Receipt		X							
Labor & Delivery Characteristics					X				
Infant Weight, Head Circumference					X			X	
Maternal Vital Status			X		X				X
Maternal Morbidity			X		X				X
Maternal Nasal Swab									X If indicated
Maternal Pregnancy Related Morbidity				X					
Maternal Temperature				X	X				
Maternal Weight, Blood Pressure, Pulse	X			X					
Infant Vital Status					X				X
Infant Morbidity					X				X
Infant Nasal Swab									X if indicated
Infant Temperature					X				
**Substudy #1 (Antibody Transfer)**									
Maternal pre & post vaccine blood samples	X (pre)				X (post)				
Infant cord blood sample					X				
Maternal breast milk sample						X	X	X	
**Substudy #2 (Family Transmission)**									
Other family members (OFM) vital status									X
OFM Morbidity									X
OFM Nasal Swab									X if indicated

#### Household census

A census of all households in the study area was conducted as a part of phase 1 in order to identify all married women age 15–40 years for recruitment, obtain informed consent for participation, and to collect basic information about the household and its structure. Basic information was recorded on age, date of birth, marital status, and pregnancy status for all married women of reproductive age.

#### Pregnancy Identification

Initial identification of pregnant women occurred at the baseline household visit prior to phase 1. Subsequently, all married women between 15 and 40 years of age were visited every 5 weeks and asked about their menstrual period in the preceding 5 weeks. If the woman had her period, the week of the period was recorded and she was visited again 5 weeks later. If she reported no menstrual period, she was offered a urine-based pregnancy test at her home. If the test was positive, she received a pregnancy enrollment interview that included her reproductive history, morbidity in the previous month, date of her last menstrual period, tobacco and alcohol use, and plans for where and by whom she would deliver her baby. We also measured her weight, height, blood pressure, temperature and pulse. Pregnant women were encouraged to attend antenatal care clinics in the public or private sector and to deliver at a certified birthing facility. The study provided education on nutrition, clean delivery, and danger signs at this visit. We also provided her with 90 days of iron-folic acid supplements, a clean birthing kit (included a clean blade, string, plastic tarp, and small plastic disc on which to cut the cord), chlorhexidine ointment for umbilical cord antisepsis, and a single dose of albendazole.

We visited all women monthly throughout their pregnancy to collect pregnancy-related morbidity in the previous month ask about tobacco and alcohol use, and to measure weight, blood pressure, pulse, and temperature.

At the time of pregnancy identification, information on household characteristics was collected and included measures of household socioeconomic status using an asset index, ethnicity, total number of persons in the household, house construction materials and size, source of drinking water, household sanitation practices, type of fuel used for cooking, and whether there were members of the household working outside the local area or country. A complete household roster was also completed at this time including information on age, date of birth, sex, marital status, literacy, educational level, and tobacco use for each member of the household.

#### Trial enrollment visit

Enrollment/vaccination visits occurred at a central location in the VDCs on a rotating basis. Upon arrival at the clinic site, consent to participate was reconfirmed with the woman, her TT history was collected, and a short interview was conducted to reconfirm eligibility. She was listed on the sequential vaccine receipt log and the vaccinator was provided the sealed envelope that matched her serial number. She was subsequently immunized with her assigned treatment in a private location by an unmasked study vaccinator.

#### Pregnancy outcome assessment

When a baby was born, the family contacted a local staff member as soon as possible, often during labor. Project staff then visited the home to collect extensive information on the birth process and the health of the mother and newborn infant(s). Weight, length, head circumference, and temperature of the infant(s) were measured and recorded. One week following delivery, a visit was made to record information on maternal post-partum morbidity.

#### Respiratory morbidity assessment

All women and their newborn infants were visited weekly for morbidity assessment as described previously. Women were visited during pregnancy and through 6 months post-partum. Infants were visited through 6 months of age. Once a woman and her infant reached 180 days post-partum they were discharged from the study.

#### Cause of death assessment

All women and children who died were identified and a verbal autopsy was conducted with members of the family after a culturally appropriate mourning period.

### Laboratory procedures

Mid-nasal specimens were collected using nylon flocked swabs and placed in 1.5 mL of Primestore transport medium, as described. Following local storage at ambient temperature for up to one week, tubes were shipped to the local laboratory, aliquotted into cryovials with secure seals, and stored in a refrigerator prior to shipment at room temperature to the University of Washington Molecular Diagnostics Laboratory, where the samples were stored at −80°C. For PCR analysis, total nucleic acids were extracted from 200 μL of Primestore as described for nasal washes [23] and tested for influenza A and B RNA using real-time reverse transcription (RT)-PCR targeting the influenza A and B matrix genes [24]. Specimens positive for influenza A were subtyped for the seasonal influenza A H1 and H3 subtypes and the novel 2009 H1N1 subtype using real time RT-PCR assays, which employ primers and TaqMan probes that target the specific subtype hemagglutinin genes. Extracted nucleic acid samples were added to a commercial master mix (UltraSense One-Step RT-PCR kit, Invitrogen) containing the forward and reverse primers and the probe in a final volume of 40 μL. RT-PCR cycling conditions are according to the manufacturer’s recommendations for a total of 40 cycles. Positive and negative controls consisting of uninfected human cells and clinical specimens confirmed positive for each influenza A subtype by the Washington State Public Health Laboratory were included in each extraction and PCR run. Samples were considered positive for influenza A or B if the PCR amplification plots crossed the threshold before cycle 40. Each assay had a 95% limit of detection of 10 copies per reaction or 500 copies per swab.

Assays were also conducted for other respiratory pathogens including pertussis and respiratory viruses on a subset of specimens using similar PCR techniques [24,25]. Nasal swabs for culture were collected in UTM Viral Transport Media (Copan Diagnostics, Inc., Murrieta, CA). These tubes were kept in a cooler box after collection and subsequently stored in liquid N_2_ in the field laboratory and transported to Cincinnati where they were stored at −70°C.

Maternal blood was collected using venipuncture into sterile vacutainers. These tubes were kept in a cooler box and transported to the field laboratory. Umbilical cord blood was collected in sterile urine cups by milking the cut umbilical cord. All blood samples were spun, the serum drawn off and then aliquoted prior to storing in liquid N_2_. Serum samples were transported to Cincinnati where they were stored at −70°C.

The serum analysis for antibodies was performed by the Laboratory for Specialized Clinical studies at Cincinnati Children’s Hospital. This lab is certified by the Clinical Laboratory Improvement Amendments (CLIA) and the College of American Pathologists (CAP). Sera were first assessed for antibody to each of the three components of the vaccine by hemagglutination inhibition (HAI) assay using standard methods [26-28]. In brief, sera were treated with Receptor-Destroying Enzyme (RDE; Denka-Seiken, Japan) to remove non-specific inhibitors of hemagglutination prior to testing. Following RDE treatment, the samples were further diluted to 1:10 in Phosphate Buffered Saline (PBS). The sera were then treated with turkey packed red blood cells (RBCs) to remove non-specific agglutinins. The RBCs were spun out of the sera and the samples were ready for testing. Starting at 1:10 dilution, the sera were diluted two-fold through 1:2560 in V-bottom microtiter plates.

Egg-derived, inactivated viral antigens A/California/7/2009 (H1N1)pdm09, A/Perth/16/2009(H3N2 and B/Brisbane/60/2008 (B/Victoria) representative of the vaccine from 2010 to 2012 and A/California/7/2009 (H1N1), A/Victoria/361/2011 (H3N2 and B/Wisconsin/1/2010 (from the B/Yamagata lineage of viruses) representative of the vaccine from 2012 to 2013 were obtained from the Influenza Reagent Resource (www.influenzareagentresource.org). The antigens were added to serially diluted sera, and incubated at room temperature for 30 minutes. RBCs from turkey blood (Viromed Laboratories, Minnetonka, MN) were suspended at a concentration of 0.5% in PBS, added to the serum/viral antigen mixture, and incubated at room temperature for 30 minutes. Plates were tilted and read. The antibody titers were reported as the reciprocal of the last serum dilution to completely inhibit RBC agglutination. Sera without reactivity were assigned a value of < 10. Sera with initial titers of ≥ 2560 were retested at a higher starting dilution in order to obtain a reportable titer. Control sera were established for each antigen and were run in each assay. The assay was valid if the control sera fell within 2 fold of their defined titer. The three antigens used in the assay were shown to be specific by using reference sera supplied by the CDC before use in the HAI. Microneutrilization assays were conducted by the Centers for Disease Control and Prevention according to their standard protocol [29].

### Sample size

Sample size requirements were driven by the primary outcomes for these trials.

All three primary outcomes were considered equivalent in importance and thus, the Type I error was corrected for these multiple primary endpoints. We assumed a total Type I error of 5% across the three primary endpoints, thus needing a sample size corresponding to one-third of this value, or 0.017 for each primary outcome.

1. **Laboratory Confirmed Influenza Illness in Infants 0–6 months of age:** We assumed a baseline rate of 21.6/100 person-years based on the Bangladesh trial [18]. A sample size of 1850 pregnancies (925 in each group) would detect a 50% reduction with 90% power for this endpoint.
2. **Low Birthweight:** Data on the expected incidence of low birthweight came from previous studies we have conducted in this area [30]. The current best estimate is 30.4/100 live births. With 1850 pregnancies, a 25%-30% relative reduction would be detected with 90% power.
3. **Influenza-Like-Illness in Women in Pregnancy and through 6 Months Postpartum:** We assumed a baseline rate of 34/100 person-years based on previous studies in this area [31]. With 1850 pregnancies, a 30% to 35% relative reduction in incidence was detectable with 90% power.

### Data management and analysis

Data were collected on paper forms and checked for errors and missing values before being sent from the field site to Kathmandu for data entry. Data entry screens checked for inconsistencies and out of range values. Errors were corrected by data supervisors based on all relevant forms or sent back to the field for clarification if not time sensitive data.

Two separate analyses will be conducted for each of the annual two cohorts of women vaccinated. A consort diagram will be constructed to show the number of women enrolled, randomized, pregnancy outcomes and live born infants, and losses to follow up through 6 months postpartum. A table will show baseline comparability of the two randomized groups using household and individual maternal level characteristics. The primary outcomes will be compared using relative risk ratios and 95% confidence intervals. Primary outcomes may be stratified by time periods where influenza exposure was high, moderate or low.

### Ethical review

These trials were reviewed and approved by the Institutional Review Boards at the Johns Hopkins Bloomberg School of Public Health (USA), the Institute of Medicine at Tribhuvan University (Nepal), and Cincinnati Children’s Medical Center (USA) and registered at Clinicaltrials.gov (NCT01034254). The study was also approved by the Nepal Health Research Council. An independent Data and Safety Monitoring Board (DSMB) was formed and was available to discuss emergent issues if they should arise. This group met formally once prior to the start of the study, once approximately mid-way through the first trial, and is scheduled to meet again in early 2015 to review the results of both trials.

## Discussion

We have provided a description of the design and methods used in a consecutive pair of community-based, placebo-controlled, randomized trials of influenza vaccine in pregnant women on respiratory outcomes in the mothers and their infants and in the outcomes of pregnancy conducted in rural Nepal. Interest is growing in using maternal immunization as a strategy to protect both women and their newborns from important infectious disorders, especially early prior to the normal time at which immunizations are scheduled in infancy. The most recognized model for this approach is immunization of pregnant women with tetanus toxoid for prevention of neonatal tetanus. This has proven to be highly effective and is a routine practice in almost every country [16]. As we anticipate the approvals of new vaccines, especially those against serious respiratory illness in young infants such as respiratory syncytial virus, the option of immunizing the mother to transfer protection to her infant in that vulnerable period of early infancy is becoming more attractive.
